# Breaking Dawn: The twilight of starch degradation in the light

**DOI:** 10.1093/plphys/kiac202

**Published:** 2022-05-06

**Authors:** Sarah Courbier

**Affiliations:** Centre for Integrative Biological Signalling Studies (CIBSS), University of Freiburg, Schänzlestraße 18, 79104 Freiburg, Germany; Institute of Biology II, University of Freiburg, Schänzlestraße 1, 79104 Freiburg, Germany

Plants have the amazing ability to convert solar energy into sugars through photosynthesis. Photosynthesis-derived sugars, synthesized during the day, serve as building blocks for a myriad of metabolic and developmental processes in plants (i.e. respiration, cell wall biosynthesis, amino acids synthesis, and the production of secondary metabolites). Upon synthesis, these sugars can either be used right away or converted into starch to be used at night, during which starch reserves are remobilized and degraded into simple sugars (i.e. maltose), allowing plants to maintain growth until photosynthesis resumes the next day (Stitt and Zeeman, 2012). Usually, starch synthesis and degradation are assumed as two separate processes occurring either during the day (starch synthesis) or during the night (starch degradation). However, it is long known that starch degradation can occur in the light as well (Stitt and Heldt, 1981; Lu et al., 2005; Weise et al., 2006). More recent studies even demonstrated that the rate of starch degradation in the light increases as the duration of light exposure increases (Fernandez et al., 2017). Although the fact that starch degradation also occurs in the light has been observed for decades, its biological relevance for plant growth and its regulation remain unclear.

In this issue of *Plant Physiology*, Ishihara et al. (2022) further characterized the biological relevance of starch degradation in the light. They demonstrate that starch degradation in the light is regulated via processes similar to those operating at night. Also, they elegantly show that starch degradation in the light is mediated by the sucrose-sensor molecule trehalose 6-phosphate (Tre6P), which regulates and stabilizes carbon resources during evening twilight.

To investigate how light impacts starch homeostasis, the authors measured starch accumulation kinetics in response to changes in photoperiod. First, they used plants entrained in short (6-h photoperiod)- or long (18-h photoperiod)-day conditions prior to being transferred to a continuous light regime on the day of the experiment. After transfer to continuous light, both short and long day-entrained plants displayed near-linear starch accumulation before plateauing at ZT = 14 h (ZT; Zeitgeber time = 14 h after the lights were turned on). Even though short day-entrained plants expected dusk at ZT = 6 h, they continued to accumulate starch reserves similarly as in long day plants, indicating that starch accumulation depends on the time after dawn, irrespective of time to expected dusk.

Then, Ishihara et al. (2022) focused on starch accumulation dynamics in response to a decrease in irradiance imposed at different times of the photoperiod (to simulate evening twilight). When applied a few hours before dusk, simulated twilight barely reduced starch accumulation in short day plants while it caused a net decrease in starch levels in long day plants, showing that the impact of twilight on starch degradation is relative to the time elapsed since dawn (ZT = 0 h), regardless of the remaining time to dusk. When imposed early in the day, simulated twilight hardly affected starch accumulation in long day plants. Similarly to simulated twilight, a decrease in CO_2_ levels had a similar impact on reducing starch accumulation, pointing toward the involvement of photosynthesis rather than light signaling components.

**Figure 1:**
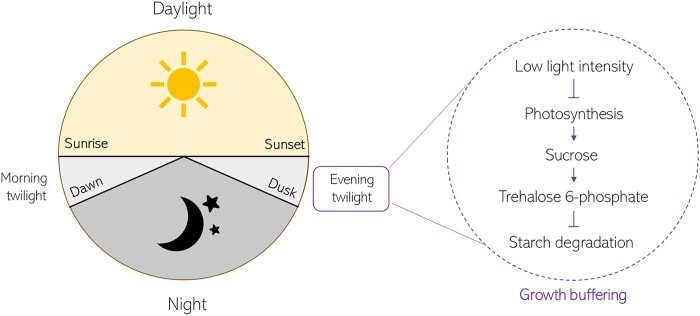
Proposed mechanism of how evening twilight impacts starch degradation. The authors proposed that low light intensity during evening twilight reduces photosynthesis activity, thereby reducing levels of the sucrose-sensing molecule Tre6P, which usually inhibits starch degradation. Starch degradation during twilight allows plants to maintain and buffer growth when photosynthesis declines to avoid an energy crisis shortly before dusk. Figure based on the findings from Ishihara et al. (2022).

Next, to test whether starch degradation in the light could be regulated like starch degradation occurring in the night, the authors modified and optimized an existing arithmetic division model (Scialdone et al., 2013) to predict starch degradation rate (and starch content) upon transfer of the plants to continuous light. The model predicted a gradual decrease in starch accumulation during the day leading to net loss of starch shortly before dusk (due to a higher starch degradation rate in the evening). This prediction was contradicted by their physiological data showing, not a decrease but, a stabilization of starch levels before dusk. Using ^13^CO_2_ labeling, the authors showed that the stabilization of starch resources was associated to the maintenance of cell wall and protein synthesis upon simulated dusk twilight. Based on these results, the authors hypothesized that decreased light intensity during evening twilight causes a dampening of photosynthesis and the associated sucrose/starch accumulation, which would in turn trigger starch degradation to maintain starch levels constant until dusk and avoid an energy crisis (Figure 1).

To test their hypothesis, Ishihara et al. (2022) investigated the role of Tre6P in regulating starch accumulation in the light. Tre6P is a sucrose sensor molecule known to regulate starch homeostasis by inhibiting starch degradation (Figueroa and Lunn, 2016). By using ethanol-inducible Tre6P synthase-overexpressing lines (displaying increased levels of Tre6P upon induction by ethanol), the authors showed that increased Tre6P levels corresponded with increased starch levels, corroborating the idea that Tre6P inhibits starch degradation.

Altogether, the authors provide evidence that decreased light intensity during evening twilight reduces photosynthesis activity and subsequent sucrose/starch synthesis, which in turn decreases Tre6P levels in plants, thereby promoting starch degradation to maintain starch levels constant until dusk (Figure 1). This increased starch degradation during evening twilight allows plants to buffer growth processes when photosynthesis efficiency declines.

Tre6P is a key regulator of starch degradation during the night as it integrates the circadian clock to set the pace at which starch must be degraded to sustain plant growth until the next day (dos Anjos et al., 2018). The authors speculate that the clock could also play a role in pacing starch degradation in the light, too. This idea would be an interesting follow-up study to confirm whether starch degradation in the light could simply be the reverse as when it occurs at night. Indeed, the fact that starch levels are barely affected by simulated twilight applied early in the day could underlie adaptive mechanisms of plants to distinguish between simple weather fluctuations (i.e. sun flecks or clouds) when compared with evening twilight. In addition, evening twilight is usually associated with cooler temperatures, which would be an interesting parameter to model and study in the context of the regulation of starch degradation in the light. In conclusion, Ishihara et al. (2022) beautifully combine model predictions with physiological data to further our understanding of starch degradation in the light and its biological and ecophysiological relevance.

*Conflict of interest statement*. None declared.
